# The i148m *Pnpla3* polymorphism influences serum adiponectin in patients with fatty liver and healthy controls

**DOI:** 10.1186/1471-230X-12-111

**Published:** 2012-08-16

**Authors:** Luca Valenti, Raffaela Rametta, Massimiliano Ruscica, Paola Dongiovanni, Liliana Steffani, Benedetta Maria Motta, Elena Canavesi, Anna Ludovica Fracanzani, Enrico Mozzi, Giancarlo Roviaro, Paolo Magni, Silvia Fargion

**Affiliations:** 1 Department of Internal Medicine, Università degli Studi Milano, UO Medicina Interna 1B, Fondazione IRCCS Ca’ Granda Ospedale Maggiore Policlinico, Milan, Italy; 2 Department of Endocrinology, Pathophysiology and Applied Biology, Università degli Studi Milano, Milan, Italy; 3 Department of Surgery, Università degli Studi Milano, Ospedale Maggiore “Ca’ Granda” Fondazione Policlinico IRCCS, Milan, Italy

**Keywords:** Adiponectin, Adiponutrin, Chronic hepatitis C, Fibrosis, Gender, Genetics, Nonalcoholic fatty liver disease, Nonalcoholic steatohepatitis, Pnpla3, Steatosis

## Abstract

**Background:**

Reduced adiponectin is implicated in the pathogenesis of nonalcoholic fatty liver disease (NAFLD) and steatohepatitis (NASH), and the I148M *Patatin-like phospholipase domain-containing 3* (*PNPLA3*) polymorphism predisposes to NAFLD and liver damage progression in NASH and chronic hepatitis C (CHC) by still undefined mechanisms, possibly involving regulation of adipose tissue function. Aim of this study was to evaluate whether the I148M *PNPLA3* polymorphism influences serum adiponectin in liver diseases and healthy controls.

**Methods:**

To this end, we considered 144 consecutive Italian patients with NAFLD, 261 with CHC, 35 severely obese subjects, and 257 healthy controls with very low probability of steatosis, all with complete clinical and genetic characterization, including *adiponectin (ADIPOQ)* genotype. *PNPLA3* rs738409 (I148M) and *ADIPOQ* genotypes were evaluated by Taqman assays, serum adiponectin by ELISA. Adiponectin mRNA levels were evaluated by quantitative real-time PCR in the visceral adipose tissue (VAT) of 35 obese subjects undergoing bariatric surgery.

**Results:**

Adiponectin levels were independently associated with the risk of NAFLD and with the histological severity of the disease. Adiponectin levels decreased with the number of 148 M *PNPLA3* alleles at risk of NASH both in patients with NAFLD (p = 0.03), and in healthy subjects (p = 0.04). At multivariate analysis, *PNPLA3* 148 M alleles were associated with low adiponectin levels (<6 mg/ml, median value) independently of NAFLD diagnosis, age, gender, BMI, and *ADIPOQ* genotype (OR 1.67, 95% c.i. 1.07-2.1 for each 148 M allele). The p.148 M *PNPLA3* variant was associated with decreased adiponectin mRNA levels in the VAT of obese patients (p < 0.05) even in the absence of NASH. In contrast, in CHC, characterized by adiponectin resistance, low adiponectin was associated with male gender and steatosis, but not with *PNPLA3* and *ADIPOQ* genotypes and viral features.

**Conclusions:**

The I148M *PNPLA3* variant is associated with adiponectin levels in patients with NAFLD and in healthy subjects, but in the presence of adiponectin resistance not in CHC patients. The I148M *PNPLA3* genotype may represent a genetic determinant of serum adiponectin levels. Modulation of serum adiponectin might be involved in mediating the susceptibility to steatosis, NASH, and hepatocellular carcinoma in carriers of the 148 M *PNPLA3* variant without CHC, with potential therapeutic implications.

## Background

Paralleling the epidemics of obesity and the metabolic syndrome (MetS), nonalcoholic fatty liver disease (NAFLD) has become the leading cause liver disease in Western countries, affecting 16-34% of the general population, and a higher proportion of overweight subjects [1]. When complicated by steatohepatitis (NASH), liver damage can progress to cirrhosis and hepatocellular carcinoma [2]. NAFLD is defined by an excess of liver triglycerides in the absence of excessive alcohol intake or exposure to toxics, and is metabolically characterized by insulin resistance (IR), which is more severe in the presence of NASH [3-6]. Increased lipolysis due to adipose tissue IR is key to NAFLD pathogenesis [5,7], and is associated with altered release of adipokines, and in particular adiponectin, a molecule with insulin-sensitizing, anti-inflammatory, and anti-fibrotic effects [8], whose decreased levels have been shown to correlate with hepatic fat accumulation [9-11]. Adipose tissue IR is increased, and adiponectin levels decreased, in patients with severe NASH compared to healthy controls independently of body mass, and correlate with histological improvement after treatment [12]. Overall evidence suggests also the existence of a correlation between adiponectin levels and progressive hepatocellular damage [13]. Defective adiponectin activity has also been demonstrated in chronic hepatitis C (CHC), but in this case the mechanism seems related to adiponectin resistance, leading to hyper-adiponectinemia, especially in patients with severe fibrosis [14]. However, decreased adiponectin has been associated with steatosis also in CHC [15].

The I148M polymorphism of *Patatin-like phospholipase domain-containing 3* (*PNPLA3*), influences hepatic triglycerides accumulation and the susceptibility to fibrotic NASH in adults and children [16-19], steatosis susceptibility and liver damage progression in CHC [19-22], and progression to hepatocellular carcinoma [21]. Although the I148M variant directly interferes with hepatic lipid metabolism [23-25], it has also been reported to influence adipocytes size and leptin transcription in obese children with NAFLD [26], and in mice deletion of *Pnpla3* influenced gene expression in adipose tissue [25], thus suggesting that modulation of adipose tissue function might be involved in the pathogenesis of liver disease associated with the I148M polymorphism. However, no data are available on the effect of I148M *PNPLA3* variant on adiponectin levels.

The aim of this study was therefore to assess whether the I148M *PNPLA3* variant influences adiponectin in patients with liver diseases characterized by altered adiponectin activity and whose disease progression is influenced by this genetic variant and steatosis, i.e. NASH and CHC, and in healthy controls with a very low probability of steatosis. To increase the power of the study, the analysis was controlled for *adiponectin* (*AdipoQ*) genotype, which is the major inherited determinant of adiponectin levels [27,28] and was reported to predispose to steatosis in non-obese non-diabetic subjects [29].

## Methods

### Patients

We considered 144 consecutive unrelated patients of Italian ancestry followed at the Metabolic Liver Diseases outpatient service, Fondazione IRCCS Ca’ Granda Ospedale Maggiore Policlinico, with a new diagnosis of NAFLD (without cirrhosis) between January 2008 and January 2010, whose serum (stored at −80°C) and DNA samples were available. One hundred fifteen patients underwent liver biopsy because of persistently abnormal liver enzymes/serum ferritin or a long lasting history of steatosis associated with severe metabolic abnormalities. Other causes of liver disease were excluded, including increased alcohol intake (>30/20 g/day for M/F), and carboxydesialylated transferrin determination, viral and autoimmune hepatitis, hereditary hemochromatosis, and alpha1-antitrypsin deficiency.

We considered 261 Italian patients with CHC, all with biopsy, followed at the Metabolic Liver Diseases outpatient service, who were included in a previous study [21], and for whom a serum sample collected at the time of liver biopsy and stored at −80°C was still available.

The control group included 257 blood donors from Northern Italy who were selected because of availability of serum samples stored at −80°C, lack of clinical and biochemical evidence of liver and metabolic disease, and no alcohol abuse (<30/20 g/day in males/females). We excluded subjects with ALT > 35/30 IU/ml in males/females, GGT > 35 IU/ml, BMI > 28, abdominal circumference > 100 cm, glucose levels ≥ 100 mg/dl, triglycerides ≥ 150 mg/dl, HDL ≤ 45/55 in M/F or a fatty liver index > 35, a value with high specificity to rule out NAFLD in the general population [30]. The study protocol was approved by the Institutional Review Board of the Fondazione IRCCS Ca’ Granda IRCCS. Informed written consent was obtained from each patient and control subject, and the study conforms to the ethical guidelines of the 1975 declaration of Helsinki. Demographic and clinical features are shown in Table 1. 

**Table 1:** **Demographic, clinical, histological, metabolic features, and*PNPLA3*I148M genotype of NAFLD and CHC patients, and healthy controls with very low probability of steatosis**

	**NAFLD patients (n = 144)**	**CHC patients (n = 261)**	**Healthy controls (n = 257)**
Sex (F)	32 (22%)	110^ (42%)	55 (21%)
Age (years)	49.5±12	58.1±14^	48±12
BMI (Kg/m^2^)	26.8±3.3^	25.4±3.9	25.0±2.8
Ferritin (ng/ml)	470±420^	248±372^	52±47
Total cholesterol (mg/dl)	206±40	178±38^	193±33
HDL cholesterol (mg/dl)	47±13^	50±17^	57±13
Triglycerides (mg/dl)	133±64^	105±50^	88±46
Glucose (mg/dl)	98±27^	95±23^	67±39
Diabetes	17 (12%)^	47 (18%)^	0
ALT (IU/ml)	60±46^	70±59^	22±9
GGT (IU/ml)	87±122^	61±69^	21±13
Steatosis	144 (100)^	179 (69)^	0
Steatosis 2-3	41* (36)^	23 (9)^	0
NASH	52* (45)	NA	NA
Fibrosis	60* (52)	258 (99)	NA
Cirrhosis	0	80 (31)^	0
FFAs (mmol/l)°	0.22 ± 0.01^	NA	0.16±0.01
Insulin (IU/ml)°	14.3 {10.8-20}^	NA	11.7 {9.2-14.5}^
HOMA-IR°	3.2 {2.5-4.8}^	NA	2.5 {1.9-3.2}
Adipo-IR°	14.9±14.5^	NA	6.1±2.7
Adiponectin (μg/ml)	4.9 {3.0-7.8}^	9.4 {5.7-13.3}^	6.6 {4.7-9.3}
*ADIPOQ*			
rs2241766TT and rs1501299T+	83 (57)	143 (55)	142 (55)
rs2241766G + or rs1501299GG	61 (43)	118 (45)	115 (45)
*PNPLA3* rs738409			
CC (148 I/I)	55 (38)	133 (51)	146 (57)
CG (148 I/M)	68 (47)	103 (39)	95 (37)
GG (148 M/M)	21 (15)	25 (10)	16 (6)

*BMI* body mass index, *HOMA-IR* homeostasis model assessment insulin resistance index, *NASH* nonalcoholic steatohepatitis, *FFAs* free fatty acids, *Adipo-IR* adipose tissue insulin resistance index, *NA* not addressed, ():% values, *n = 115 patients with liver biopsy, *adiponectin* (*ADIPOQ*) genotype was classified according to [29], available in all patients and 68 healthy controls, ^p < 0.05 vs. controls.

### Histological assessment

Tissue sections were stained with hematoxylin and eosin, impregnated with silver for reticulin framework, and stained with trichrome for collagen. One expert pathologist unaware of clinical and genetic data reviewed all biopsies for fibrosis stage. The severity of steatosis, features of NASH and fibrosis was assessed according to Kleiner et al. [31]. NASH was considered to be present when steatosis, ballooning and lobular inflammation were present. In CHC patients, liver histology was assessed according to Ishak [32]. Liver steatosis was also scored according to Kleiner [31]. The minimum biopsy size was 1.7 cm and the number of portal areas 10.

### Adiponectin measurement

All the evaluations have been performed on an aliquot of plasma collected after overnight fasting collected at the time of liver biopsy (when available) of the abdominal ultrasonography and metabolic evaluation, which allowed the diagnosis of NAFLD, and stored at −80°C. Plasma adiponectin levels (all isoforms) were measured using a commercial enzyme-linked immunosorbent assay kit with a lowest limit of sensitivity of 0.246 ng/mL (R&D Sytems, Minneapolis, MN) [33]. Insulin levels were evaluated in all patients and 68 healthy controls by the DRG Insulin ELISA kit (DRG International, Mountainside, NJ). Serum FFAs were also evaluated in all patients and 68 healthy controls by the Free fatty acid quantification kit (Biovision Research products, Mountain View, CA). Briefly, FFAs are converted to their CoA derivatives, which are subsequently oxidized with the concomitant generation of color or fluorescence. C-8 (octanoate) and longer fatty acids generated can then be easily quantified by colorimetric (spectrophotometry at λ = 570 nm) methods. The adipose tissue insulin resistance (Adipo-IR) index was calculated by multiplying fasting FFAs x insulin (mmol/l / pmol/l) [34].

### Adiponectin mRNA evaluation

In 35 consecutive patients who underwent gastric banding because of morbid obesity (age 45±10 years, 5 males, BMI 41.9±7 Kg/m^2^, 16 with NASH), protocol wedge biopsies of omental visceral adipose tissue (VAT) were performed as a part of a research protocol for which approval of the Ethical Committee and written informed consent were obtained. All patients underwent also liver biopsy at the time of surgical procedure, and all had liver steatosis. Total RNA was isolated by the Trizol reagent (Lifetech, Carlsbad, CA), digested with DNaseI, and quality evaluated by measuring the 260/280 nm absorbance ratio (≥ 1.8) and by electrophoresis. First-strand cDNA was synthesized using equal amounts (0.5 μg) of total RNA, with the SuperScript VILO cDNA synthesis kit (Invitrogen, Carlsbad, CA). Adiponectin mRNA levels were analyzed by qRT-PCR with SYBR Green chemistry (Fast SYBR Green master mix, Applied Biosystems, Foster City, CA). All the reactions were performed in triplicate with the ABI 7500fast quantitative real time (qRT)-PCR system (Lifetech, Carlsbad, CA). Primers are available upon request. Results were normalized for beta actin and HPRT1, which were chosen as a control because of stable expression among different samples, and reported as arbitrary units (i.e. fold expression relative to normalized adiponectin expression of a control sample used as internal standard for calibration of qRT-PCR reactions).

### Genetic analysis

DNA was extracted from peripheral blood by the phenol-chloroform method. The *adiponutrin/PNPLA3* rs738409 C > G SNP, encoding I148M, was genotyped by a Taqman assay (assay on demand for rs738409, Applied Biosystems, Foster City, CA) by personnel unaware of patients and controls clinical status. Post-PCR allelic discrimination was carried out measuring allele-specific fluorescence on the Opticon2 detection system (MJ Research, Waltham, MA) [17]. Adiponectin (*ADIPOQ* ) rs2241766, +45 G > T and r150299, +276 G > T polymorphisms, previously associated with the susceptibility to NAFLD [29], were also genotyped by a Taqman assay (assay on demand, Applied Biosystems, Foster City, CA).

### Statistical analysis

Results are expressed as means ± standard deviation for normally distributed variables, median {interquartile range} for non-normally distributed variables (e.g. adiponectin), which were log transformed before analysis. Mean values were compared by Anova or Wilcoxon, when appropriate, and frequencies by Fisher’s exact test. Variables were correlated by the Spearman’s rho test. Independent predictors of the presence of NAFLD, NASH, and fibrosis, and of low adiponectin levels were determined by logistic regression analysis. Analyses were carried out with the PASW 18.0 (SPSS - IBM, Chicago, IL) and the JMP 6.0 (SAS, Cary, NJ) statistical analysis software.

## Results

### Adiponectin levels are associated with NAFLD

As expected, despite similar sex distribution, patients with NAFLD had higher BMI, prevalence of dyslipidemia, IR, increased liver enzymes NAFLD, serum insulin (p < 0.0001), FFAs (p < 0.0001), HOMA-IR (p < 0.0001), Adipo-IR (p < 0.0001), and lower adiponectin levels (p = 0.008) compared to healthy controls with very low probability of steatosis (Table 1). At multivariate logistic regression analysis conducted in patients and controls with complete metabolic data, FFAs (OR 1.013, 95% c.i. 1.007-1.018; p < 0.0001) and adiponectin (OR 0.870, 95% c.i. 0.794-0.953; p = 0.003) were associated with NAFLD independently of age, sex, BMI, glucose, and insulin levels (Table 2).

**Table 2:** Independent demographic, anthropometric, and metabolic predictors of the presence of NAFLD at logistic regression analysis in 212 subjects from Northern Italy with complete metabolic data

	**OR**	**95% c.i.**	**p**
Age (years)	1.016	0.98-1.05	0.342
Sex (M)	2.211	0.84-5.81	0.108
BMI (Kg/m^2^)	1.368	1.17-1.60	<0.0001
Glucose (mg/dl)	0.999	0.97-1.03	0.959
Insulin (IU/ml)	1.173	1.00-1.38	0.055
FFAs (mmol/l)	1.013	1.01-1.02	<0.0001
Adiponectin (μg/ml)	0.870	0.79-0.95	0.003

*OR* odds ratio, *c.i*. confidence interval, *BMI* body mass index, *FFAs* free fatty acids.

### Adiponectin, steatosis, and liver damage in subjects with NAFLD

Adiponectin levels were correlated with serum FFAs (rho = −0.141; p = 0.043), Adipo-IR (rho = −0.155; p = 0.026), ALT levels (rho = −0.411; p < 0.0001), severity of steatosis (rho = 0.200; p = 0.033), necroinflammation (rho = −0.297; p = 0.001), NAFLD activity score (rho = −280; p = 0.003), and fibrosis stage (rho = −0.297, p = 0.001), and was inversely correlated with fasting insulin (rho = −0.156, p = 0.002). Independent predictors of steatosis grade 2–3, NASH, and the presence of fibrosis in biopsied patients are shown in Table 3. Adiponectin levels were associated with a reduced risk of moderate/severe steatosis (OR 0.83, 95% c.i. 0.72-0.96), NASH (OR 0.86, 95% c.i. 0.75-0.98), and of fibrosis (OR 0.84, 95% c.i. 0.74-0.95) independently of age, sex, BMI, ALT, insulin, glucose levels, and FFAs.

**Table 3:** Independent predictors of steatosis grade 2–3, NASH, and of fibrosis at logistic regression analysis in 115 consecutive biopsied patients with NAFLD with available DNA and serum samples at the time of liver biopsy

**Variables**	**Steatosis grade 2–3 vs. 1**		**NASH vs. simple steatosis**		**Fibrosis vs. no fibrosis**	
	**OR (95% c.i.)**	**p value**	**OR (95% c.i.)**	**p value**	**OR (95% c.i.)**	**p value**
Age (years)	1.04 (0.99-1.09)	0.094	1.01 (0.96-1.06)	0.598	1.06 (1.01-1.10)	0.020
Sex (F)	1.31 (0.40-4.2)	0.656	0.88 (0.27-2.89)	0.834	0.55 (0.18-1.65)	0.282
BMI (Kg/m^2^)	1.22 (1.04-1.44)	0.014	1.32 (1.11-1.56)	0.002	1.13 (0.99-1.30)	0.079
ALT (IU/ml)	1.02 (1.01-1.03)	0.007	1.01 (1.00-1.02)	0.028	1.01 (1.00-1.02)	0.028
Glucose (mg/dl)	0.99 (0.98-1.01)	0.217	1.03 (1.00-1.05)	0.061	1.02 (0.99-1.04)	0.214
Insulin (IU/ml)	1.01 (0.97-1.06)	0.618	0.99 (0.94-1.04)	0.654	0.98 (0.94-1.03)	0.473
FFAs (mmol/l)	1.00 (0.98-1.01)	0.453	1.00 (0.99-1.01)	0.671	1.00 (0.99-1.01)	0.934
Adiponectin (μg/ml)	0.83 (0.72-0.96)	0.012	0.86 (0.75-0.98)	0.025	0.84 (0.74-0.95)	0.006

*OR* odds ratio, *c.i.* confidence interval, *BMI* body mass index, *FFAs* free fatty acids; the presence of NASH and fibrosis were defined according to the NASH clinical research network definition [31].

### PNPLA3 genotype influences adiponectin levels in subjects without viral hepatitis

The frequency distribution of the I148M *PNPLA3* variant was significantly different between patients and controls (p < 0.0001; Table 1), due to an over-representation of the 148 M variant in patients, whereas *ADIPOQ* genotype was not significantly associated with NAFLD (in both male and females). Clinical features of patients and controls with very low probability of steatosis subdivided according to *PNPLA3* I148M are shown in Table 4. *PNPLA3* I148M genotype was associated with adiponectin levels both in patients (p = 0.03) and in controls (p = 0.04), whereas it did not influence other metabolic features.

**Table 4:** **Clinical features of 144 NAFLD patients and 257 healthy controls with very low probability of steatosis from Northern Italy according to the*PNPLA3*I148M genotype**

	**NAFLD patients (n = 144)**			p value*
*PNPLA3* genotype	CC (n = 55)	CG (n = 68)	GG (n = 21)	
Age (years)	49±13	51±12	47±12	0.87
Sex (F)	15 (27)	12 (18)	5 (24)	0.58
BMI (Kg/m^2^)	26.8±3.7	27.1±3.1	26.0±3.0	0.62
ALT (IU/ml)	54±38	58±49	80±54	0.06
Diabetes	3 (5.5)	11 (16.2)	3 (14.3)	0.13
Insulin (IU/ml)	14.6 {11.7-19.9}	13.4 {10.2-20.2}	16.8 {11.5-22.1}	0.33
Glucose	94±21	101±34	97±16	0.43
HOMA-IR	3.1 {2.5-4.5}	3.3 {2.4-4.5}	3.3 {2.4-5.5}	0.57
FFAs (mmol/l)	0.21±0.09	0.22±0.08	0.22±0.09	0.85
Adipo-IR	14.2±15.1	13.9±13.9	17.9±15.4	0.78
Adiponectin (μg/ml)	5.5 {3.0-9.9}	4.9 {3.0-7.4}	3.8 {2.7-6.5}	0.03
	**Controls (n = 257)**			p value*
*PNPLA3* genotype	CC (n = 146)	CG (n = 95)	GG (n = 16)	
Age (years)	46±12	51±12	45±14	0.51
Sex (F)	37 (25)	15 (16)	3 (19)	0.83
BMI (Kg/m^2^)	24.8±2.7	25.2±2.8	25.2±4.2	0.71
ALT (IU/ml)	21±7	24±8	22±6	0.48
Insulin° (IU/ml)	11.8 {9.5-14.0}	11.9 {9.2-15.4}	9.0 {7.6-14.8}	0.17
Glucose	89±6	89±6	86±3	0.89
HOMA-IR°	2.5 {1.9-3.2}	2.6 {2.0-3.4}	1.8 {1.7-3.0}	0.10
FFAs° (mmol/l)	0.16±0.07	0.14±0.06	0.23±0.02	0.93
Adipo-IR°	6.4±2.8	5.2±2.1	8.1±0.9	0.47
Adiponectin (μg/ml)	7.0 {4.7-9.9}	6.2 {4.7-8.8}	5.6 {4.2-7.4}	0.04

* p for trend across *PNPLA*3 genotypes, °available in 68 controls. *BMI* body mass index, *IFG* impaired fasting glucose, *HOMA-IR* homeostasis model assessment insulin resistance index, *FFAs* free fatty acids, *Adipo-IR* adipose tissue insulin resistance index, ():% values, {}: interquartile range.

**Figure 1:**
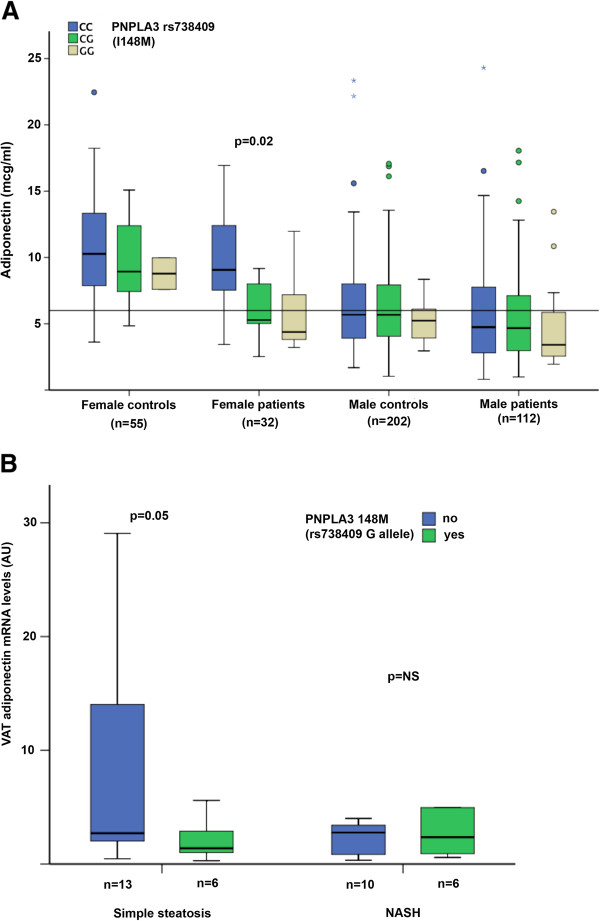
**Effect of*PNPLA3*genotype on serum adiponectin.A**) Adiponectin levels according to I148M *PNPLA3* genotype in subjects stratified according to gender and NAFLD diagnosis vs. healthy controls. **B**) Adiponectin mRNA levels (AU: arbitrary units) in VAT of morbidly obese patients according to the presence of NASH and I148M *PNPLA3* genotype.

In contrast, *ADIPOQ* genotype was associated with adiponectin levels in controls (7.6±3.8 in rs2241766TT and rs1501299T + vs. 6.8±3.8 μg/ml in rs2241766G + or rs1501299GG; p = 0.048), but not in patients with NAFLD (not shown). The difference was more marked in males (6.9±3.5 in rs2241766TT and rs1501299T + vs. 5.8±3.4 μg/ml in rs2241766G + or rs1501299GG; p = 0.021), whereas it was not significant in females (not shown). Adiponectin levels in subjects subdivided according to *PNPLA3* genotype after stratification for gender and the presence of NAFLD are shown in Figure 1A. In this subgroup analysis, the I148M variant was significantly associated with adiponectin in females with NAFLD.

In the whole series of subjects without CHC (Table 5), low adiponectin levels (lower than 6 μg/ml, median value in controls) were associated with the presence of NAFLD, age, male gender, BMI, dyslipidemia, ALT, insulin levels, *PNPLA3* I148M alleles (p = 0.01), and *ADIPOQ* genotype (p = 0.009). Interestingly, the 148 M *PNPLA3* allele was associated with low serum adiponectin in subjects carrying the *ADIPOQ* rs2241766G + or rs1501299GG “low adiponectin” genotypes (50, 47% 148I/I; 64, 65% I/M, 20, 80%, M/M; p = 0.0018), but not in those with the rs2241766TT and rs1501299T + “high adiponectin” genotypes (47, 46% 148I/I; 30, 45% I/M, 6, 46%, M/M; p = 0.99). At logistic regression analysis (Table 5), low adiponectin levels were associated with NAFLD diagnosis, younger age, male sex, higher BMI, *ADIPOQ* genotype, and *PNPLA3* 148 M alleles (OR 1.67, 95% c.i. 1.07-2.64 for each 148 M allele carried). In patients with histological NAFLD, *PNPLA3* 148 M alleles were associated with low adiponectin levels independently of age, sex, BMI, *ADIPOQ* genotype, and the severity (grade 1 to 3) of steatosis (OR 2.20, 95% c.i. 1.09-4.80; p = 0.027).

**Table 5:** Variables associated with and independent predictors of low adiponectin levels (<6 mg/ml) in 144 NAFLD patients and 257 healthy controls with very low probability of steatosis

**Adiponectin**	**Low (n = 217)**	**High (n = 194)**	**p**	OR * (95% **c.i.)**	p *
NAFLD present	89 (46)	52 (24)	0.002	1.65 (1.02-2.70)	0.043
Age (years)	46±11	51±13	0.007	0.96 (0.94-0.98)	<0.0001
Sex (F)	20 (9)	67 (34)	<0.0001	0.21 (0.11-0.37)	<0.0001
BMI (Kg/m^2^)	26.2±3.0	25.0±3.1	<0.0001	1.15 (1.07-1.25)	0.0002
HDL (mg/dl)	48±12	57±14	<0.0001	-	-
Triglycerides (mg/dl)	119±68	92±47	<0.0001	-	-
ALT (IU/ml)	42±38	29±25	<0.0001	-	-
Insulin (IU/ml)	17±14	13±8	0.003	-	-
FFAs (mmol/l)	0.21±0.09	0.19±0.08	0.05	-	-
*ADIPOQ*					
rs2241766TT and rs1501299T+	83 (38)	100 (52)	0.008	0.61 (0.39-0.95)	0.030
rs2241766G + or rs1501299GG	134 (62)	94 (48)		reference	
*PNPLA3* rs738409					
CC (148 I/I)	97 (45)	112 (58)	0.01	1.67° (1.07-2.64)	0.024
CG (148 I/M)	94 (43)	71 (37)			
GG (148 M/M)	26 (12)	11 (6)			

():% values; * OR of low adiponectin at logistic regression analysis considering as independent variables the presence or not of NAFLD, age, gender, BMI, *ADIPOQ* and *PNPLA3* genotype (additive model; OR per number of variant 148 M alleles).

### Effect of I148M PNPLA3 on adiponectin expression in VAT

As expected, in severely obese patients VAT adiponectin mRNA levels tended to be lower in patients with (n = 16) than in those without (n = 19) NASH (2.9±2.9 vs. 5.5±5.8 arbitrary units (AU); p = 0.1). In this series adiponectin was not significantly associated with gender (possibly due to the very small number of males included), age, and BMI. Adiponectin mRNA levels according to *PNPLA3* genotype and NASH are shown in Figure 1B. The presence of the 148 M variant was associated with lower adiponectin even in the absence of NASH (2.2±2, n = 10 vs. 9.0±10, n = 9; p = 0.050).

### Effect of PNPLA3 genotype on adiponectin levels in CHC patients

Clinical features of CHC patients subdivided according to *PNPLA3* genotype are presented in Table 6. *PNPLA3* genotype was associated with steatosis (p = 0.02), severe steatosis (p < 0.0001), and nearly associated with cirrhosis (p = 0.06), but was not significantly associated with metabolic features.

**Table 6:** **Clinical features of 261 CHC patients from Northern Italy according to the*PNPLA3*I148M genotype**

***PNPLA3*genotype**	**CC n = 133 (51)**	**CG n = 103 (39)**	**GG n = 25 (10)**	**p value**
Age (years)	57±14	59±14	57±14	0.47
Sex (F)	58 (44)	46 (45)	6 (24)	0.16
BMI (Kg/m^2^)	25.2±3.4	25.3±3.9	26.6±6.1	0.25
ALT (IU/ml)	72±56	67±58	73±78	0.81
Diabetes	26 (20)	17 (17)	4 (16)	0.80
Insulin (IU/ml)	21 {14–27}	20 {17–27}	17 {12–25}	0.61
Glucose	95±22	96±26	93±22	0.85
Total cholesterol (mg/dl)	176±36	177±41	178±41	0.95
HDL cholesterol (mg/dl)	50±15	51±18	48±15	0.66
Triglycerides (mg/dl)	108±43	104±61	88±31*	0.24
HOMA-IR	4.7 {3.2-5.8}	4.5 {3.4-6.6}	3.8 {2.3-6.5}	0.62
Adiponectin (μg/ml)	9.3 {5.8-13}	9.4 {5.7-14}	9.7 {5.3-13}	0.93
Steatosis	85 (64)	72 (70)	22 (88)	0.02
Severe steatosis (> 33%)	8 (6)	7 (7)	8 (32)	<0.0001
Cirrhosis	34 (26)	36 (35)	10 (40)	0.06

():% values; {}: interquartile range, reported with median value; *p < 0.05 vs. other genotypes.

Adiponectin levels were higher in subjects with CHC (with and without steatosis) than in those without CHC (NAFLD patients and healthy controls): 9.4 {5.7-13.2} vs. 5.8 {3.9-8.9} μg/ml; p < 0.0001. At multivariate logistic regression analysis, the association between adiponectin and CHC was independent of age, gender, BMI, the presence of steatosis, and *ADIPOQ* genotype (OR 1.10, 95% c.i. 1.03-1.19; p = 0.0009). Variables associated with low adiponectin levels in CHC patients are shown in Table 7. Low adiponectin levels were associated with steatosis, age, male gender, dyslipidemia, cirrhosis, and viral genotype 4 (patients carrying genotype 4 were all males), nearly associated with *ADIPOQ* genotype (p = 0.068), but not with *PNPLA3* I148M genotype (p = ns). However, there was an association between the 148 M/M genotype low adiponectin levels in subjects carrying the “high adiponectin” rs2241766TT and rs1501299T + genotypes (6, 46% vs. 20, 19%; p = 0.026). There was no significant association between adiponectin and viral load, other viral genotypes, and the histological activity of the disease (not shown). At logistic regression analysis (Table 7), low adiponectin levels were associated with male sex, younger age, and steatosis, but not with *PNPLA3* and *ADIPOQ* genotypes.

**Table 7:** Variables associated with and independent predictors of low adiponectin serum levels (<6 μg/ml) in 261 CHC patients

**Adiponectin**	**Low (n = 72)**	**High (n = 189)**	**p**	OR * (95% **c.i.)**	p *
Steatosis present	59 (82)	120 (63)	0.004	3.85 (1.78-8.91)	0.0005
Age (years)	51±14	61±13	<0.0001	0.95 (0.92-0.97)	<0.0001
Sex (F)	10 (14)	100 (53)	<0.0001	0.18 (0.08-0.38)	<0.0001
HDL (mg/dl)	42±11	53±17	<0.0001	-	-
Triglycerides (mg/dl)	121±71	98±38	0.012	-	-
Cirrhosis	15 (21)	65 (34)	0.036	0.65 (0.30-1.40)	0.277
Viral genotype 4	11 (15)	8 (4)	0.0055	1.68 (0.55-5.70)	0.456
*ADIPOQ*					
rs2241766TT and rs1501299T+	26 (36)	92 (49)	0.068	0.74 (0.39-1.40)	0.355
rs2241766G + or rs1501299GG	46 (64)	97 (51)		reference	
*PNPLA3* rs738409					
CC (148 I/I)	34 (47)	99 (52)	0.73	1.15 (0.71-2.80)	0.600
CG (148 I/M)	30 (42)	73 (39)			
GG (148 M/M)	8 (11)	17 (9)			

():% values; * OR of low adiponectin values at logistic regression analysis considering as independent variables the presence or not of NAFLD, age, gender, BMI, ALT, and *ADIPOQ* and *PNPLA3* genotype (additive model; OR per number of 148 M alleles).

## Discussion

As decreased adiponectin, a molecule which antagonizes hepatic lipid accumulation and has insulin-sensitizing, anti-inflammatory, and anti-fibrotic effects [8] plays a pivotal role in NAFLD pathogenesis [9-11,13], and the I148M *PNPLA3* polymorphism involved in the regulation of lipid metabolism predisposes to the progression of liver diseases associated with steatosis by still undefined mechanisms, we evaluated whether *PNPLA3* genotype influenced adiponectin levels. Our results support the hypothesis that *PNPLA3* genotype is a determinant of serum adiponectin in patients with NAFLD, in particular in females, and in healthy controls, independently of acquired factors, and of *ADIPOQ*[27,28].

We preliminarily confirmed that patients with NAFLD displayed lower adiponectin than healthy controls [13]. Although we did not exclude steatosis by imaging in all control subjects, the metabolic and clinical selection criteria used have a high specificity to exclude this condition in the general population [30], and the different distribution of *PNPLA3* genotype between patients and healthy subjects with very low probability of steatosis indirectly confirmed the accurate selection of controls [16,17]. Moreover, in patients with NAFLD low adiponectin was independently associated with the severity of steatosis, as well as with NASH and fibrosis, in line with the hypothesized role of adiponectin in the progression of liver damage [9,11,13,35].

The I148M *PNPLA3* variant is a major risk factor for the development and progression of NAFLD [16-18,22,36], but the mechanism linking the 148 M allele to increased liver fat, inflammation, and fibrogenesis, and the tissue specificity of PNPLA3 physiological activity are still under definition [25,37]. The 148 M *PNPLA3* allele was previously associated with adipocytes of smaller size, a marker of metabolic derangement, and increased leptin transcription in a small group of obese children with NAFLD [26], thus suggesting that modulation of adipose tissue endocrine activity may be implicated in the mechanism by which *PNPLA3* influences NASH susceptibility. In the present study, we could not detect a significant association between *PNPLA3*, FFAs, and insulin resistance [36], whereas in line with the experimental hypothesis *PNPLA3* genotype influenced serum adiponectin levels. Indeed, we observed a dose dependent relationship between the 148 M variant and adiponectin, which was evident in both in healthy controls and patients with NAFLD, but, due to the very well known gender-specific dimorphism of adiponectin levels which are higher in females [8], it more frequently contributed to hypo-adiponectinemia in females. Interestingly, these data may contribute to explain the significant interaction between *PNPLA3* I148M genotype and female gender on NAFLD risk observed in the recent meta-analysis by Sookoian et al. [36]. The observation that even in the absence of NASH severely obese patients carrying the 148 M variant had decreased VAT adiponectin mRNA expression is also in line with these findings and suggests that the reduced serum adiponectin associated with this genetic polymorphism is determined by impaired transcription of the gene in the adipose tissue. However, given the relatively limited number of subjects analyzed (in particular of obese subjects), the relationship between *PNPLA3* genotype and adiponectin, and the lack of association with IR should be confirmed in further studies. Indeed, recent studies have reported an association of the *PNPLA3* I148M variant with IR and lower levels of triglycerides in severely obese subjects [38,39], suggesting that in the presence of environmental triggering factors PNPLA3 is indeed a modulator of hepatic IR and lipoprotein secretion.

Furthermore, although the association between *PNPLA3* genotype and adiponectin was independent of steatosis severity in patients with NAFLD, our results does not exclude that *PNPLA3* may modulate adipose tissue indirectly by regulating the secretion of hepatokines such as fetuin [40], and that it may also modulate other pro-inflammatory molecules such as resistin and ICAM-1 [26,41].

The genetic determinants of serum adiponectin levels, a strongly heritable trait, have been recently evaluated in genome-wide association studies [27,28]. Although no association with *PNPLA3* has emerged so far, only a small fraction (<10%) of adiponectin heritability has been determined, and none of the studies considered patients with NAFLD. The strongest genetic determinant of adiponectin levels is *ADIPOQ*[27,28], and *ADIPOQ* SNPs have been associated with steatosis and progressive NASH in lean patients without diabetes and dyslipidemia [29]. In this study, we characterized *ADIPOQ* genotype, which allowed to adjust the results for a major confounder and to unveil a possible interaction between *ADIPOQ* and *PNPLA3* in determining adiponectin levels. The lack of association between *ADIPOQ* and NAFLD risk may be explained by the different inclusion criteria in our study compared to the previous [29].

On the other hand, despite in line with previous findings *PNPLA3* genotype influenced steatosis in CHC and was possibly associated with lower triglycerides, in these patients *PNPLA3* and *ADIPOQ* were not significantly associated with adiponectin, possibly due to development of adiponectin resistance associated with chronic HCV infection, in particular in patients with more severe disease [14]. Adiponectin resistance could modify the interaction between genotype and environmental factors, decreasing the effect size of genetic variants, so that the power of the present study was not sufficient to detect significant differences in adiponectin levels. This conclusion is supported by our data indicating that CHC is independently associated with higher adiponectin, previously ascribed to decreased adiponectin receptor-1 mediated clearance [14], in particular in the presence of advanced fibrosis [14]. In contrast, although cirrhotic patients were not included in the NAFLD group, adiponectin levels decreased with the severity of liver damage in patients with steatosis, in line with the results of a recent meta-analysis [13].

## Conclusions

In conclusion, our data suggest that the I148M *PNPLA3* genotype may represent a genetic determinant of serum adiponectin levels. Modulation of serum adiponectin might be involved in mediating the susceptibility to steatosis, NASH, and hepatocellular carcinoma in carriers of the 148 M *PNPLA3* variant without CHC, with potential therapeutic implications, but additional studies are required to confirm this hypothesis.

## Abbreviations

Adipo-IR : Adipose tissue insulin resistance index; ALT : Alanine transaminase; AST : Aspartate transaminase; BMI : Body mass index; c.i. : Confidence interval; FFAs : Free fatty acids; GGT : Gamma-glutamyl transferase; HOMA-IR : Homeostasis model assessment insulin resistance index; IR : Insulin resistance; MetS : Metabolic syndrome; NAFLD : Nonalcoholic fatty liver disease; NAS : Nonalcoholic fatty liver disease activity score; NASH : Nonalcoholic steatohepatitis; PNPLA3 : Patatin like phospholipase domain containing −3; SNP : Single nucleotide polymorphism; VAT : Visceral adipose tissue.

## Competing interests

The authors declare that they have no competing interest to disclose.

## Authors’ contribution

LV designed the study, analyzed and interpreted the data and wrote the manuscript draft. RR, BMM, and PD performed genetic and gene expression analyses and contributed to data analyses, MR, LS, and PM measured serum adiponectin and contributed to data analysis, EC contributed to data analysis and to manuscript drafting, EM and GCR collected and processed tissue samples and contributed to data analysis, AF collected clinical data, SF contributed to data interpretation and supervised the study. All Authors read and approved the final manuscript version.

## Authors’ information

Luca Valenti is assistant professor of Internal Medicine at the Metabolic Liver Diseases Center, directed by prof. S. Fargion, of the Università degli Studi di Milano, Fondazione Ca’ Granda IRCCS Ospedale Maggiore Policlinico. He coordinates a research team contributing to this paper, and his main research interests include metabolic liver diseases, and in particular nonalcoholic fatty liver disease and hereditary hemochromatosis, iron metabolism, and the role of genetics in hepatology. The research group has recently contributed to unravel the role of PNPLA3 in the progression of liver damage in liver diseases associated with steatosis. He has established collaborations with prof. Giancarlo Roviaro, Department of Surgery Università degli Studi di Milano, for the study of genetic factors influencing hepatological complications in severily obese patients undergoing bariatric surgery, and with Paolo Magni and Massimiliano Ruscica, Department of Endocrinology, Pathophysiology and Applied Biology, Università degli Studi Milano, for the study of endocrinological and metabolic alterations associated with nonalcoholic fatty liver disease.

### Financial support

The work was supported by the following grants: FIRST Università degli Studi di Milano 2007, 2008 (LV, SF: http://www.unimi.it); Ricerca corrente Ospedale Maggiore Policlinico 2006 and 2008 (LV, SF; http://www.policlinico.mi.it); and Centro per lo Studio delle Malattie del Fegato e del Metabolismo. The funders had no role in study design, data collection and analysis, decision to publish, or preparation of the manuscript.

## Pre-publication history

The pre-publication history for this paper can be accessed here:

http://www.biomedcentral.com/1471-230X/12/111/prepub
